# Efficacy of cryotherapy for the treatment of cutaneous leishmaniasis: meta-analyses of clinical trials

**DOI:** 10.1186/s12879-016-1663-3

**Published:** 2016-07-26

**Authors:** Liliana López-Carvajal, Jaiberth Antonio Cardona-Arias, María Isabel Zapata-Cardona, Vanesa Sánchez-Giraldo, Iván Darío Vélez

**Affiliations:** 1 University of Antioquia, Calle 62 No. 62-59, Lab 632, Medellin, Colombia; 2 School of Microbiology, University of Antioquia UdeA, Calle 70 No. 52-21, Medellín, Colombia; 3 School of Medicine, Cooperative University of Colombia, Medellín, Colombia; 4 Health and Sustainability Research Group, School of Microbiology and Bioanalysis, University of Antioquia, Medellín, Colombia

**Keywords:** Efficacy, Cryotherapy, Leishmaniasis, cutaneous, Clinical trials as a topic, Meta-analysis as a topic

## Abstract

**Background:**

Cryotherapy is a local treatment for cutaneous leishmaniasis with variable efficacy and greater safety than conventional treatment. The objective of this study is to evaluate the efficacy and safety of cryotherapy for the treatment of cutaneous leishmaniasis and to compare it with pentavalent antimonials.

**Methods:**

A meta-analysis based on a search of nine databases with eight strategies was conducted. Inclusion and exclusion criteria were applied, the methodological quality of each article was evaluated, and the reproducibility of the study selection and information extraction from each clinical trial was assured. The per lesion and per patient efficacy was calculated, and a meta-analysis of relative risks with the random effects model and the Dersimonian and Laird's, Begg, and Egger tests, along with a sensitivity analysis, were performed. A meta-regression based on the methodological quality of the trials included was also performed.

**Results:**

Eight studies were included in which respective per lesion efficacies of 67.3 % and 67.7 % were reported for cryotherapy and pentavalent antimonials. In 271 patients treated with cryotherapy and in 199 with pentavalent antimonials, respective per protocol and intent to treat efficacies of 63.6 % and 54.2 % were found in the first group, and per protocol and intent to treat efficacies of 74.7 % and 68.3 % were found in the second group. The relative risk for the comparison of efficacy in the two groups was 0.73 (0.42–1.29). The results of the sensitivity analysis and the meta-regression analysis of relative risks were statistically equal to the overall results.

**Conclusion:**

This investigation provides evidence in favor of the use of cryotherapy given that its efficacy is similar to that of pentavalent antimonials.

**Electronic supplementary material:**

The online version of this article (doi:10.1186/s12879-016-1663-3) contains supplementary material, which is available to authorized users.

## Background

Leishmaniasis is a parasitic group of diseases produced by protozoans, belonging to the *Leishmania* spp. genus, and transmitted by dipterous insects of the *Phlebotomus* spp. genus in the old world and *Lutzomyia* spp. in the new world. Its reservoirs are wild and domestic animals, and the disease has a great clinical polymorphism that can be grouped into three large categories: Cutaneous Leishmaniasis (CL), Mucosal Leishmaniasis (ML) and Visceral Leishmaniasis (VL) [1]. In the old world, leishmaniasis is caused chiefly by *Leishmania major, L. tropica, L. aethiopica, L. donovani* and *L infantum*. In the new world, it is chiefly caused by *L. braziliensis, L. panamensis, L. guyanensis, L. amazonensis, L. peruviana, L. mexicana* and *L. infantum* [2, 3].

Approximately 0.7 to 1.2 million cases of CL are contracted each year. Approximately one-third of these cases are contracted in the Americas, the basin of the Mediterranean and western Asia, from the Middle East to Central Asia. In total, 70 to 75 % of the world incidence of CL is reported in the countries of Afghanistan, Algeria, Colombia, Brazil, Iran, Syrian, Ethiopia, North Sudan, Costa Rica and Peru [4].

Specifically, CL affects the skin and mucous membranes, with lesions characterized as being painless, lone or multiple, ulcerated, verrucous or in the shape of nodules, and located on the face, neck, arms and legs [2]. This clinical presentation consequently carries a social stigma, making the patient feel excluded and humiliated. The disease can also impact their interpersonal relationships and psychological health, with women being the most affected [5]. In Afghanistan, some mothers with CL have prohibited their children from being touched to avoid infecting them, and in Colombia, it has been reported that cutaneous ulcers in a woman can be the pretext for their husband's rejection [6].

Leishmaniasis is a neglected tropical disease and has strong links with poverty. The risk of infection increases in the presence of unhealthy conditions and poor environmental sanitation and malnutrition and in the absence of personal protection measures. In addition, the lack of access to health care systems causes delays in diagnosis and appropriate treatment, increasing morbidity and mortality. The cost of treatment for leishmaniasis is high ($ 30 to $ 1500 per medication), which is considered to be a factor impacting the poverty of the families affected [6].

Treatment for leishmaniasis seeks to prevent morbidity and mortality (especially for VL), to accelerate clinical and parasitological healing, to reduce scars, to prevent relapses and dissemination, and to avoid resistance to the medications utilized [7]. In the last 70 years, pentavalent antimonials have been the first therapeutic option. However, due to their adverse effects, other intravenous and oral alternatives have been implemented. Generally, treatment depends on medical criteria and is based on the clinical-epidemiological evaluation of the patient, the country of origin, the species prevalent in the specific location and the levels of adaptation to available alternatives in each community [8].

The pentavalent antimonials include N-methylglucamine antimoniate (Glucantime®) and sodium stibogluconate (Pentostam®), for which high frequencies of secondary effects have been reported. Prior reports indicate adverse events, such as myalgia, arthralgia, abdominal pain, queasiness and, in some cases, increases in amylase, pancreatic lipase, liver transaminases, renal failure, leukopenia, anemia, thrombocytopenia and cardiotoxicity [8, 9]. For that reason, these medications are contraindicated in patients who present damage in any of these related systems, in expectant or nursing mothers, and in older adults. Other therapeutic alternatives include local treatment, utilized chiefly for patients with few and small lesions. Local treatment is usually less toxic and is better tolerated than systemic treatment [8].

Faced with the limitations described for pentavalent antimonials, cryotherapy has been studied as a treatment for CL, as it is a fast procedure that does not usually present secondary effects or contraindications [10]. This stimulus produces decreases in the local tissue temperature and metabolism [11], which results in cryonecrosis that destroys the amastigotes and activates an immune response produced by the liberation of antigenic substances [12]. This response reduces the risk of secondary hypoxia in the tissue adjacent to the lesion [11]. In spite of these advantages, the available evidence with regard to the degree of efficacy of this treatment compared to pentavalent antimonials for CL is inconclusive. In this regard, some studies have reported efficacies as low as 20.0 % [13] and as high as 93.3 % [14]. In addition, clinical studies that have compared cryotherapy with pentavalent antimonials have had low sample sizes, possibly affecting the statistical power of the results [13, 15].

This evidence highlights the need to develop a meta-analysis of the efficacy of cryotherapy, given that this methodology offers the necessary techniques to efficiently and rigorously collect the results of studies on the same subject matter. In addition, a meta-analysis can synthesize the available scientific information, increase the validity of the conclusions from individual studies, and analyze sources of heterogeneity in the results. A meta-analysis can also explain possible contradictions between results on a similar topic, thus identifying areas of uncertainty where it is necessary to conduct further research [16].

The objective of the present study was to evaluate the efficacy and safety of cryotherapy in the treatment of CL and to compare it with pentavalent antimonials.

## Methods

### Type of study

Systematic review with a meta-analysis of clinical trials.

### Patient (P)/Intervention (I)/Comparison (C)/Outcome (O) (PICO) question

#### Population

Patients with a diagnosis of CL, parasitologically confirmed by swab or culture, or a clinical-histopathological diagnosis.

#### Intervention

Cryotherapy performed with two or more treatment cycles with a duration of between 10 and 30 seconds per lesion in each session, according to the type of lesion [8].

#### Comparison

Pentavalent antimonials available on the market. Specifically, sodium stibogluconate (Pentostam® or generic) or meglumine antimoniate (Glucantime® or generic), which are chemically similar, show the highest quality of evidence and are highly recommended for the treatment of CL [17].

##### Outcome

Proportion of patients healed, with healing defined as complete re-epithelialization of the injury, leveling of the active border of the lesion, disappearance of the base induration, absence of new lesions and disappearance of mucosal involvement in cases where it was presented [17].

### Research Protocol according to Preferred Reporting Items for Systematic Reviews and Meta-Analyses (PRISM) guidelines [18]

#### Identification of search strategy

A search of articles in the following bibliographical databases was performed: EBM Reviews – CCRCT, Embase, Lilacs, OVID, PubMed, ScienceDirect, Scielo, Web of Science and Wiley – based on the following search strategy: i) "Cutaneous leishmaniasis" and "Treatment", ii) "Cutaneous leishmaniasis" and "Topical treatment", iii) "Cutaneous leishmaniasis" and "Local treatment", iv) "Cutaneous leishmaniasis" and "Local heat", v) "Cutaneous leishmaniasis" and "Heat therapy", vi) "Cutaneous leishmaniasis" and "Thermal therapy", vii) "Cutaneous leishmaniasis" and "Cryotherapy" and viii) "Cutaneous leishmaniasis" and "Cryosurgery". Some of the syntax utilized were: i) (cutaneous leishmaniasis[Title/Abstract]) AND cryotherapy[Title/Abstract], ii) (cutaneous leishmaniasis and cryotherapy). mp. [mp = title, abstract, original title, name of substance word, subject heading word, keyword heading word, protocol supplementary concept word, rare disease supplementary concept word, unique identifier], iii) TITLE-ABSTR-KEY (cutaneous leishmaniasis AND cryotherapy), iv) (ti:(Cutaneous leishmaniasis)) AND (ab:(cryotherapy)). In addition, a search was made in google scholar, without obtaining additional results to those found with other searches.

#### Screening or application of inclusion criteria

i) Articles that include the search terms in the title and abstract, ii) original studies, iii) research performed in humans, and iv) manuscripts for which primary outcomes involved the evaluation of the efficacy of cryotherapy for the treatment of cutaneous leishmaniasis. The search was not restricted by language or publication year.

#### Election or application of the exclusion criteria

i) Descriptive, cross-sectional and prospective studies that only evaluated one group treated with cryotherapy, ii) studies in which the cryotherapy was combined with other substances and the result of the monotherapy was not reported, iii) clinical trials in which the cryotherapy was compared against placebo or medications that were different from pentavalent antimonials, such as ketoconazole, metronidazole and imiquimod, and iv) studies with small sample sizes (*n* < 15).

### Reproducibility and evaluation of the methodological quality of the studies included

Three investigators independently applied the study protocol to guarantee the reproducibility of the study selection. Discrepancies were resolved by consensus and referral to the other two investigators. To guarantee the reproducibility of the extraction of information, a form was produced in Excel with the study variables and was independently filled out by each of the three investigators. After this, each of the three files in excel was blinded and a fourth researcher did the analysis of reproducibility. Kappa indices were calculated for the variables of the data extraction protocol: author, title, year, place of study, population, exclusion, specie, time of following, the methodological characteristicper and protocol and intent to treat therapeutic efficacies and methodological quality. In all cases, a value of 1.00 was obtained.

In the evaluation of the methodological quality of the studies, a form with the following criteria was completed: randomization, concealment and blinding, calculation of the sample size, application of the inclusion and exclusion criteria, intent to treat analysis, report of safety data and analysis of homogeneity by variables such as number, type, location and size of lesion, prior history of cutaneous leishmaniasis, species of the parasite, development time of the disease and other information reported in the studies, such as sociodemographic conditions. These criteria were coded as zero if they were incomplete and one if they did not have explicit information. In the data extraction there was concordance of 100 % between researchers, so the quality score was done by consensus. A summary was then made in which scores equal to or lower than 4 were considered low quality and scores of 5 or higher were considered good quality. This classification was employed for the meta-regression or analysis of two subgroups: meta-analysis of studies of low quality and other for studies with good quality.

### Statistical analysis

The articles were described according to the year of publication, the type of patients included, the causal species and the follow-up time. The per lesion and per patient efficacies were calculated, for which the proportion of healing in the two study groups was estimated. Particularly in studies that reported per patient efficacy, a per protocol and intent to treat analysis was performed. These proportions were compared with 95 % confidence intervals (CIs) for the difference of proportions with the Z statistic.

A meta-analysis of relative risks for per lesion and per patient efficacies was performed with the random effects model given the heterogeneity between studies. In the analysis of heterogeneity the Dersimonian and Laird's, Statistical Q (Chi-Square) test was performed. The RI coefficient or proportion of total variance due to the between-study variance and a Galbraith plot was also performed. Publication bias was evaluated with a Funnel Plot, and Begg's test Z Statistic and Egger's Test t Statistic were calculated. In addition, a sensitivity analysis was conducted to evaluate the effect or weight of each of the studies on the overall result. The results are reported in the form of a Forest plot and a cumulative meta-analysis. In Additional file 1: Table S1 are available all data of the meta-analysis to estimate the effectiveness of cryotherapy compared with antimonial, in an analysis (intent to treat) per patient cured.

A meta-regression based on the methodological quality of the clinical trials was performed to determine if the exclusion of low-quality studies affected the overall conclusion of the meta-analysis. The statistical power of the relative risks from the overall meta-analysis and the meta-analysis excluding low-quality studies was calculated to determine if the absence of significant differences in its conclusions were "real" or products of a decrease in statistical power (increase in β-error).

The meta-analysis of the efficacy of the per patient cryotherapy was conducted per protocol and by intent to treat. The analyses were performed in Epidat 3.1 with a significance of 0.05.

## Results

### Identification of studies

Table 1 shows the number of studies identified using the eight search strategies within eight databases and with the two initial filters that had the search terms in the title or abstract and were classified as experimental. In addition to these findings, a search was conducted in EBM Reviews – CCRCT, restricting the search to "Cutaneous leishmaniasis" in the title; 226 results were found.

**Table 1:** Absolute frequency of articles identified in the identification and screening phases

Source	Cutaneous leishmaniasis AND							
	Treatment	Topical treatment	Local treatment	Local heat	Heat therapy	Thermal therapy	Cryotherapy	Cryosurgery
PubMed								
No limits	3404	228	184	11	46	26	77	24
Title/Abstract	1197	62	15	5	7	10	52	11
Experimental	175	6	2	2	2	4	13	2
OVID								
No limits	2390	7767	11601	4990	7646	12513	4557	3938
Title/Abstract	19	9820	10579	5708	5469	7196	0	1
Experimental	5	14	12	13	13	12	0	1
ScienceDirect								
No limits	6647	1309	2863	1065	1216	57	308	169
Title/Abstract	343	34	19	4	4	4	13	1
Experimental	47	12	3	3	3	4	2	0
Embase								
No limits	2249	423	167	15	61	70	20	37
Title/Abstract	154	53	13	3	5	8	0	9
Experimental	48	34	10	3	4	8	0	2
Wiley								
No limits	3749	1220	1941	781	830	32	424	265
Title/Abstract	73	7	1	1	3	0	11	6
Experimental	47	5	1	1	3	0	5	4
Scielo								
No limits	125	32	19	1	0	1	1	0
Title/Abstract	32	2	0	0	0	1	1	0
Experimental	12	2	0	0	0	1	1	0
Lilacs								
No limits	381	230	181	20	54	32	82	26
Title/Abstract	49	73	11	4	8	1	32	9
Experimental	5	19	3	1	2	1	11	0
Web of Science								
No limits	1590	200	96	14	30	21	55	17
Title/Abstract	339	43	7	3	8	11	18	2
Experimental	95	22	3	3	5	6	12	1

**Fig. 1:**
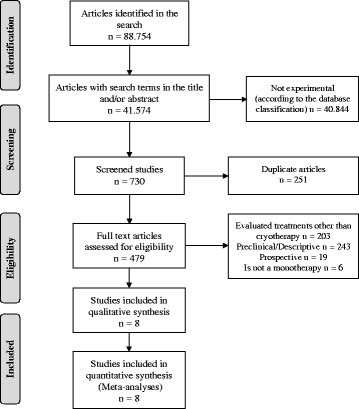
Article selection algorithm

In the initial search, 41,574 studies with the terms in the title, abstract or in both were identified. Of these, 730 were screened and classified as experimental studies from original sources, which were reduced to eight experimental studies after the exclusion of the studies that did not evaluate cryotherapy or that were preclinical experimental, descriptive or prospective studies (Fig. 1).

### Methodological quality

In the evaluation of the quality of the eight studies included, it was determined that in the Özgöztaslis O [19] and Negera E [14] studies, no explicit allusion to randomization was made; therefore, they were assumed to be quasi-experimental studies. The Salmanpour R [15] and the Negera E [14] studies did not present group homogeneity results. Only the Layegh P [10] and Thicket J [13] studies explicitly stated the criteria for the sample size calculation, and only the López P [20] study was double-blind. In general, of the seven points evaluated in the methodological quality, it was determined that the lower quality studies were those of Özgöztaslis O [19] and Negera E [14], while the higher quality studies were those of López P [20] and Thicket J [13]. It should be clarified that these criteria were evaluated as presence or absence. Because of these definitions, even studies with the same methodological quality score presented important differences in the number of items that delimited their inclusion, exclusion and analysis of homogeneity.

### Description of studies, patients, intervention and controls

Studies conducted between 1997 and 2013 were included. Two studies were from South America, four were from Asia, one was from Europe, and one was from Africa. The species corresponded to the most prevalent species in each study zone. In addition, there was high variability in the inclusion–exclusion criteria applied in each study (Table 2). In the majority of studies, treatment with pentavalent antimonials was intralesional, with the exception of the Negera study [14], in which treatment was intramuscular.

**Table 2:** Description of the studies included

Author	Year	Country	Population	Exclusion	Specie	Time
Özgöztaslis O [19]	1997	Turkey	Patients between 2–50 years seen in a medical center; mean of 12.	Sickness that is exacerbated by the cold, infected lesions and pregnant or nursing women	*Leishmania* spp.	6 months
Gurei M [22]	2000	Turkey	Patients with a confirmed parasitological diagnosis	N.E.	*N.E*	3 months
Asilian A [21]	2004	France	Patients from the Leishmania and Skin diseases Research Center. Age ranged 2–65 years, mean of 26.	Lesions > 2 months (to exclude any natural self-healing during follow-up), allergy to antimonials, those pregnant and breastfeeding	*L. tropica/L. major*	6 months
Salmanpour R [15]	2006	Iran	Patients attending the dermatological unit of the Faghihi Hospital, Shiraz	N.E.	*Leishmania* spp.	≥2 months
Layegh P [10]	2009	Iran	Patients ≤ 13 years from the Dermatological Clinic of the Qaem Hospital, Mashad. Age mean 6.8 ± 3.4 in cruytherapy and 6.2 ± 3.4 in Glucantime	Lesions ≥ 3 months, allergy to antimonials, patients who utilized another treatment at the beginning of the study	*Leishmania* spp.	6 months
López P [20]	2010	Colombia	Patients < 10 years from local hospitals in Santander and Tolima. 38.8 % women and 61.2 % men.	Use of anti-*Leishmania* drugs in the last 3 months, > 5 lesions, lesions on the border (<2 cm) of mucosal areas, eyes, nose, mouth or genitals	*Leishmania (V.) panamensis*	3 months
Negera E [14]	2012	Ethiopia	Patients from the Kibet Health Center. Age mean 18.4 ± 11.7 in cryotherapy and 19.6 ± 7.4 in SSG	N.E.	*Leishmania aethiopica*	6 months
Soto J [13]	2013	Bolivia	Patients > 12 years from the province of Chapare, Bolivia	Lesions > 30 mm, use of anti-*Leishmania* therapy in the last 3 months, history of concomitant diseases, mucosal lesions in the nose and mouth and immunosuppression	*L. braziliensis*	6 months

N.E. The article does not give explicit information about this variable

In the interventions were assessed several populations like children [10, 20], patients with a age mean of 12 and ranged 2–50 [19]; others with age mean 18.4 ± 11.7 [14] or 26 ± 11 [13] in cryotherapy group, and 19.6 ± 7.4 [14] or 29 ± 13 [13] in pentavalent antimonials group [14]; while the study of Lopez P et al. included 25 patients <18 year; 61 with age between 19–50 years and 11 higher of 50 years in cryotheraphy group, and 16 patients <18 year; 57 with age between 19–50 years and 6 higher of 50 years in pentavalent antimonials group [20]. The proportion of men and women differed among studies; some included a greater number of women [19], others included higher proportion of men [14, 20] and in other studies that proportion was similar [10, 21].

All studies applied the cryotherapy with liquid nitrogen (−195 °C), until the healthy skin 1–2 mm around the lesion appeared frozen or white; with double freeze-thaw cycle and postoperative care included daily cleansing with an antiseptic solution, except study of Negera E et al. that applied 3–4 sessions per lesion per visit [14]. Variations were found in the frequency and type of application, in one study freezing time was 30–60 seconds (s) at three week intervals [19], in others they were 10–25 s [21], 5-20s [13], 10–30s with a thawing interval of 20s [14, 15], 10–15 s with at interval of 20s, repeated weekly for 6 weeks [10]; in days 1 and 14 [13] or during 20 days [20] or 2 months [22].

The control presented different applications, intralesional meglumine antimonate (MA) every other day for 3 weeks (ten injections) [19]; intralesional pentostam injection [22];

MA injected into each lesion from all directions until the lesion had completely blanched with 0.2–1.5 cm3 per lesion per week, depending on the size [15], intralesional glucantime (Glucantime® ; Specia, Paris, France) 0.5–2 cm3 depending on lesion size injected until the lesion was completely infiltrated [10, 21], Glucantime 20 mg/kg/day for 20 days [20] or 30 consecutive days with a maximum of 850 mg/day [14] and intralesional Sb N-methylglucamine (Glucantime Rhodia Laboratories, France) 81 mg/mL on each of days 1, 3, and 5 [13].

### Efficacy analyses by lesion and patient

Three studies reported efficacy by the number of lesions: 67.3 % for cryotherapy and 67.7 % for the pentavalent antimonial (Table 3), with no statistically significant differences between the groups (Z Statistic Z = 0.020. *p*-value = 0.984). The meta-analysis of per lesion efficacy detected between-study heterogeneity, although no publication bias was present. According to the random effects analysis, no differences in the efficacy of cryotherapy and pentavalent antimonials were found. Additionally, in the sensitivity analysis, it was corroborated that none of the studies presented a greater weight in the conclusion of the meta-analysis, as the elimination of each study generated relative risks whose intervals included the null value (Table 4).

**Table 3:** Efficacy (proportion of patients cured) in the study groups

Per lesion	Cryotherapy *N* = 327 (Protocol)		Control *N* = 257 (Protocol)	
Özgöztaslis O [19]	93 (53/57)		69.7 (23/33)^a^	
Gurei M [22]	78 (47/60)		92 (67/73)^b^	
Asilian A [21]	57.2 (120/210)		55.6 (84/151)^a^	
Overall (95 % CI)	67.3 (62.0 – 72.5)		67.7 (61.8 – 73.6)	
Per patient	Per Protocol *N* = 231	Intent to treat *N* = 271	Per Protocol *N* = 182	Intent to treat *N* = 199
Salmanpour R [13]	67.8 (14/20)		75 (15/20)^a^	
Layegh P [10]	58.3 (21/36)	52.5 (21/40)	27.8 (10/36)^a^	25.6 (10/39)^a^
López P [20]	37.9 (25/66)	28.4 (25/88)	94.8 (73/77)^a^	81.1 (73/90) ^a^
Negera E [14]	93.3 (83/89)	80.6 (83/103)	89.5 (17/19)^b^	85.0 (17/20)^b^
Soto J [13]	20 (4/20)		70 (21/30)^a^	
Overall % (95 % CI)	63.6 (57.2 – 70.1)	54.2 (48.0 – 60.4)	74.7 (68.1 – 81.3)	68.3 (61.6 – 75.1)

^a^Intralesional meglumine antimonial (Glucantime® or generic)

^b^Sodium stibogluconate (Pentostam® or generic)

**Table 4:** Meta-analysis of the per lesion efficacy of cryotherapy compared to pentavalent antimonials

Per lesion efficacy N = 584 Study	RR (95 % CI)	% Weight	
		Fixed	Random
Gurei M [22]	0.85 (0.73 – 0.99)	48.5	36.2
Özgóztaslis O [19]	1.33 (1.05 – 1.69)	19.5	30.0
Asilian A [21]	1.03 (0.85 – 1.23)	32.0	33.8
Overall Fixed effects	0.99 (0.89 – 1.10)		
Overall Random effects	1.04 (0.81 – 1.32)		
Sensitivity analysis Omitted study		Relative change	
Gurei M [22] *N* = 133	1.16 (0.90 – 1.50)	11.45	
Özgóztaslis O [19] *N* = 90	0.93 (0.77 – 1.11)	−10.62	
Asilian A [21] *N* = 361	1.06 (0.68 – 1.64)	1.70	
Heterogeneity			
Dersimonian and Laird's, Q statistic (Chi-squared)	10.06 *p*-value = 0.006		
RI Coefficient (Proportion of total variance due to the between-study variance)	0.648		
Variance between studies	0,036		
Variance within studies	0,008		
Variation coeff. between studies	15,961		
No publication bias			
Begg’s test, Z Statistic	1.044, *p* = 0.296		

In five studies, 271 patients were treated with cryotherapy, and 199 were treated with pentavalent antimonials. In the first group, respective efficacies of 63.6 % (147/231) and 54.2 % (147/271) per protocol and by intention to treat were detected (Z Statistic = 2.04. *p*-value = 0.041). However, in the group treated with pentavalent antimonials, the respective efficacies were 74.7 % (136/182) and 68.3 % (136/199) per protocol and by intention to treat (Z Statistic = 1.26. *p*-value = 0.206) (Table 3).

Three studies were performed with three branches, with the third branch being the combination of pentavalent antimonials with cryotherapy or the use of placebo. In these studies, the findings were as follows: i) Asilian TO [21] with a combined therapy of cryotherapy and intralesional Glucantime® resulted in a per lesion efficacy of 90.9 % (120/132 lesions) in the per protocol analysis and an efficacy of 80.5 % (120/149) in the intent to treat analysis; ii) Salmanpour R [15] found a per patient efficacy of 89 % for the combination of cryotherapy with intralesional meglumine antimoniate (18/20); and iii) Thicket J [13] found an efficacy of 17 % in a subgroup with placebo (5/30).

**Fig. 2:**
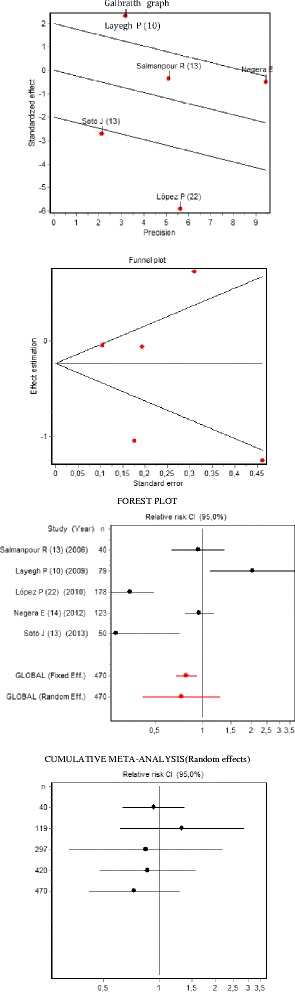
Meta-analysis of per patient efficacy of cryotherapy compared to pentavalent antimonials (intent to treat)

The meta-analysis of the per patient efficacy presented a result similar to that described for lesions, that is, the random effects analysis indicated that no statistically significant differences existed between the treatments (Fig. 2). This conclusion was obtained in both the per protocol and the intent to treat analyses. In addition, the sensitivity analysis corroborated that the elimination of each of the studies in successive steps did not modify the overall result (Table 5).

**Table 5:** Meta-analysis of the per patient efficacy of cryotherapy compared to pentavalent antimonials

Per patient efficacy study	Per protocol *N* = 413		Intent to treat *N* = 470	
	RR (95 % CI)	% Weight	RR (95 % CI)	% Weight
Salmanpour R [15]	0.93 (0.64−1.37)	21.4	0.93 (0.64−1.37)	21.5
Layegh P [10]	2.10 (1.16−3.81)	18.6	2.05 (1.11−3.77)	18.6
López P [20]	0.40 (0.29−0.55)	22.2	0.35 (0.25−0.50)	21.9
Negera E [14]	1.04 (0.88−1.23)	23.5	0.95 (0.77−1.17)	23.2
Soto J [13]	0.29 (0.11−0.71)	14.3	0.29 (0.11−0.71)	14.7
Overall random effects	0.78 (0.45−1.34)		0.73 (0.42−1.29)	
Sensitivity analysis		Relative change		Relative change
Salmanpour R [15]	0.73 (0.36−1.50)	−5.71	0.68 (0.33−1.45)	−7.04
Layegh P [10]	0.62 (0.35−1.11)	−19.9	0.58 (0.33−1.05)	−20.39
López P [20]	0.97 (0.62−1.55)	25.2	0.94 (0.59−1.48)	27.05
Negera E [14]	0.71 (0.33−1.53)	−9.3	0.67 (0.30−1.53)	−8.09
Soto J [13]	0.92 (0.52−1.61)	18.0	0.86 (0.48−1.56)	17.55
Overall random effects	0.78 (0.45−1.34)		0.73 (0.42−1.29)	
Heterogeneity				
Dersimonian- Laird's Q Statistic	42.69. *p*-value = 0.000		39.08. *p*-value = 0.000	
RI Coefficient	0.934		0.889	
Variation Coef.	4.259		2.468	
Variance between studies	0,317			0,343
Variance within studies	0,022			0,031
No publication bias				
Begg’s test, Z Statistic	0.2449. *p*-value = 0.806		0.2449. *p*-value = 0.403	
Egger’s test, t Statistic	−1.0877. *p*-value = 0.356		0.7560. *p*-value = 0.505	

*RR* (*95 % CI*) relative risk with its 95 % confidence interval

### Subgroup analysis or meta-regression

In the meta-regression, where studies were grouped according to methodological quality, the same evidence was found, which corroborates the similarity in the efficacy of cryotherapy and pentavalent antimonials. In this sense, with the exclusion of the Negera study [14] (that of lower quality), the overall relative risks were 0.71 (95 % CI = 0.33–1.53) per protocol (*N* = 305) and 0.67 (95 % CI = 0.30–1.53) by intent to treat (*N* = 347). With the elimination of this study and the Salmanpour study [15], the overall relative risks were 0.63 (95 % CI = 0.19–2.05) per protocol (*N* = 265) and 0.60 (95 % CI = 0.17–2.04) by intent to treat (*N* = 307). It should be clarified that the sensitivity analyses for the meta-regressions revealed that none of the studies altered the conclusion reported given that they all include the null value and that the exclusion of the studies with deficiencies in the design did not affect the power of the overall result (greater than 95 % for the three meta-analyses), which rules out the possibility of a β error.

### Safety analysis

The safety analysis shows multiple limitations related to the quantification of these results, given that all of the studies with the exception of Salmanpour R [15] and Layegh P [10] only list the adverse effects presented in the study groups without explicitly giving their proportions. In this sense, the most reported effects for the cryotherapy group were hypopigmentation (5.5 % in the Layegh study) [10] or reversible hyperpigmentation (19.4 % in the Layegh study) [10], erythema and edema (33 % in the Salmanpour study [15] and 100 % in the Layegh study) [10], pain and ardor. In turn, in the control group, erythema and edema, itching, ardor, fever, migraine, myalgia and arthralgia and increases in the aspartate aminotransferase (AST), alanine aminotransferase (ALT) and amylase values were reported [20].

## Discussion

The treatment of CL demonstrates the need to conduct further studies that evaluate the efficacy and safety of local treatment given the limitations of pentavalent antimonials. These limitations include frequent and serious adverse events associated with its use; contraindications or prohibited use in pregnant or nursing women and people with heart or renal alterations [20]; the long treatment duration, which does not support continued adherence; evidence of a growing decrease in therapeutic response and the need to increase the dose per kilogram of weight [23]. Along with these limitations, limitations associated with second-line treatment, such as pentamidine isethionate, Miltefosine or Amphotericin B, should also be added. These treatments may present contraindications and deleterious effects, such as nephrotoxicity and cardiotoxicity, association with persistent diabetes mellitus, pancreatitis, hypoglycemia, teratogenicity, phlebitis, anemia, and other effects. These contraindications often occur according to the medication used, which can require hospital management [8, 9].

This information demonstrates the relevance of and need for research of local treatment, particularly physical therapies such as cryotherapy that, in the case of this meta-analysis, allows us to corroborate that this strategy is an efficient therapeutic option for the treatment of CL, as it presents a proportion of healing similar to that documented for pentavalent antimonials.

It is noteworthy that this treatment can be applied without anesthesia, presents favorable results in little time, and has few secondary effects, with the majority being mild, such as reversible and transitory changes in the pigmentation of the skin [10]. To all of these advantages, the low cost of this treatment should be added (approximately $4.0 US per patient) compared to intralesional sodium stibogluconate (approximately $180.0 US per patient) [14]. In addition, some authors indicate that several species, such as *L. tropica, L. aethiopica, L. infantum* and *L. braziliensis,* are thermosensitive, which could imply that in these cases, cryotherapy could present better results [10, 21].

Although the mechanism by which cryotherapy stimulates the healing of CL is not well known, some authors have presented the hypothesis that the treatment damages infected cells through intra- and extracellular ice crystal formation, which cause alterations in the cell membrane [24]. In the parasite, temperatures below zero manage to destroy amastigotes, causing a cryonecrosis that induces an immune response as a consequence of the liberation of antigenic substances. This effect could be extended to the curative action in other lesions [12].

In spite of these findings, several limitations for the use of this therapy have been documented, such as the fact that it is not capable of immediately reaching the dermis, the layer of the skin where the infected cells are found, for which several sessions are required to eliminate the parasites without affecting the tissue [20]. Other studies exist that suggest that cryotherapy sessions with liquid nitrogen are very short or that the interval between treatments is low, reducing its potential to impede the proliferation of the parasites that survive in the lesion [25] or that it could generate relapses and therapeutic failures, while a long-term therapy could negatively impact adhesion to treatment [10, 14].

With regard to this limitation, a study conducted in Saudi Arabia reported success in patients treated with cryosurgery, confirming, through a histopathological exam, the elimination of all parasites within an hour [26]. In Jordan, cryotherapy proved to be an effective treatment for CL with one to four weekly sessions, especially for small lesions (<2 cm). Although up to three additional sessions were necessary to heal larger-sized lesions, excellent cosmetic results were obtained, with no record of relapses after a three-year follow-up [25]. This finding disputes some critiques that have been given concerning therapeutic responses to local treatment, specifically regarding cryotherapy.

In addition to what has been described, it should be clarified that the type of lesion, the progression to more serious forms or to the mucocutaneous form, and the rapidity of scar formation vary according to the *Leishmania* species [27, 28]. In this sense, some studies have shown that strains of *L. braziliensis* resistant to this type of treatment exist, for which patients infected with these strains would present significantly larger lesions compared to those infected with other species [29]. For *L. troop* and *L. major*, the variability in the energy metabolism enzymes (hexoquinase, glucose 6-phosphate dehydrogenase, lactate dehydrogenase –LDH) could affect the severity and the number of lesions. *L. troop* has a much smaller enzymatic activity than *L. major*, and the lesions produced by this species are generally more serious. However, cases exist in which the infection does not progress but can remain latent and asymptomatic or the infection may subsequently regress and conclude with scar formation of the lesion [28].

In connection with this previous point, some studies have shown that cryotherapy is an excellent therapeutic option in patients with small lesions, with a low number of lesions per patient and with a short development time (<3 months). In this context, studies have reported healing rates of 100 % in 1-cm-sized lesions and between 67 % and the 90 % in wounds with sizes between 1 and 3 cm. This criterion for treatment results in a faster clinical response in lesions with a duration less than 3 months [15].

In this context, the characteristics of the infecting species with the subsequent type of lesions that it produces are connected factors that can explain the heterogeneity in the therapeutic efficacy reported in different studies or in each endemic zone, which would support the need to generate harmonious local evidence with each clinical, parasitologic, and epidemiological profile.

Some investigators have evaluated the role of cryotherapy as a coadjuvant of pharmacological treatments, improving efficacy and reducing adverse effects while requiring a smaller dose of the medication. In this sense, the combination of cryotherapy with intralesional sodium stibogluconate (Pentostam®) has generated healing rates of up to 100 % [30]. Other authors have reported that itraconazole utilized as a monotherapy presents low efficacy (58.3 %), while its combination with cryotherapy causes an improvement (80.9 %) while reducing the risk of liver damage by the utilization of a smaller quantity of the medication [31].

In spite of the advantages of cryotherapy, other limitations of this treatment should be considered: i) some authors indicate that cryotherapy does not allow for complete healing in cases of ML; ii) some have criticized the absence of solid evidence in relation to the prevention of mucosal complications in infections caused by *L. guyanensis*, *L. panamensis* and *L. braziliensis* [8]; iii) cryotherapy is contraindicated in patients with dermatological diseases, hypertension, cardiac insufficiency or tuberculosis [32]; iv) in patients with deterioration of the immune system as newborns or older adults, a high risk of necrosis exists [25]; and v) the success of the treatment’s application in endemic zones is conditional on the availability of the specific device for the procedure and of specialized medical personnel.

In the current study, the main limitations include the low number of studies and of subjects included, the low methodological quality of some studies, the variability in the inclusion and exclusion criteria and in the homogeneity analysis, the low rigor of the safety data and the fact that meta-regression was not possible for clinical, demographic and parasitological variables. A possible bias of this review was the fact include only data published. These points highlight the need to improve reporting of the CL controlled clinical trials and to improve local evidence, adjusted for the clinical, parasitological, and therapeutic characteristics available. However, it should be clarified that these potential sources of heterogeneity in the report of the clinical trials analyzed are factors of the experimental CL study. In spite of this heterogeneity, the sensitivity analyses showed that it did not affect the overall measure reported in the current meta-analysis.

## Conclusion

Cryotherapy presents a similar efficacy to pentavalent antimonials, to which other advantages are added, such as cryotherapy having a shorter duration and better treatment adherence. This benefit could justify its utilization as a first choice treatment for CL in situations in which the pharmacological treatment is contraindicated, in areas with low frequency for ML or in patients with a history of therapeutic failure. Subsequent investigations should compare the safety and efficacy of cryotherapy with other local treatments that have shown similar results, such as thermotherapy.

## Abbreviations

CL, cutaneous leishmaniasis; ML, mucosal leishmaniasis; VL, visceral leishmaniasis

## Additional file

Additional file 1: Meta-analysis of per patient efficacy of cryotherapy compared to pentavalent antimonials (intent to treat). (XLS 29 kb)
